# Negative frequency-dependent selection or alternative reproductive tactics: maintenance of female polymorphism in natural populations

**DOI:** 10.1186/1471-2148-13-139

**Published:** 2013-07-03

**Authors:** Arne Iserbyt, Jessica Bots, Hans Van Gossum, Thomas N Sherratt

**Affiliations:** 1 Department of Biology, University of Antwerp, Evolutionary Ecology Group, Groenenborgerlaan 171, Antwerp, BE-2020, Belgium; 2 Department of Biology, Carleton University, 1125 Colonel By Drive, Ottawa, ON, K1S 5B6, Canada

**Keywords:** Alternative reproductive tactics, Colour polymorphism, Fitness, Male harassment, Odonata, Quantity-quality trade-off, Sexual conflict

## Abstract

**Background:**

Sex-limited polymorphisms have long intrigued evolutionary biologists and have been the subject of long-standing debates. The coexistence of multiple male and/or female morphs is widely believed to be maintained through negative frequency-dependent selection imposed by social interactions. However, remarkably few empirical studies have evaluated how social interactions, morph frequencies and fitness parameters relate to one another under natural conditions. Here, we test two hypotheses proposed to explain the maintenance of a female polymorphism in a species with extreme geographical variation in morph frequencies. We first elucidate how fecundity traits of the morphs vary in relation to the frequencies and densities of males and female morphs in multiple sites over multiple years. Second, we evaluate whether the two female morphs differ in resource allocation among fecundity traits, indicating alternative tactics to maximize reproductive output.

**Results:**

We present some of the first empirical evidence collected under natural conditions that egg number and clutch mass was higher in the rarer female morph. This morph-specific fecundity advantage gradually switched with the population morph frequency. Our results further indicate that all investigated fecundity traits are negatively affected by relative male density (i.e. operational sex ratio), which confirms male harassment as selective agent. Finally, we show a clear trade-off between qualitative (egg mass) and quantitative (egg number) fecundity traits. This trade-off, however, is not morph-specific.

**Conclusion:**

Our reported frequency- and density-dependent fecundity patterns are consistent with the hypothesis that the polymorphism is driven by a conflict between sexes over optimal mating rate, with costly male sexual harassment driving negative frequency-dependent selection on morph fecundity.

## Background

Evolutionary biologists have long studied visible polymorphisms as they are excellent model systems to examine micro evolutionary processes [1-3]. Polymorphisms with morphs co-existing at relatively stable frequencies appear to be common, but this phenomenon can only persist under a limited range of conditions, one of these being negative frequency-dependent selection (NFDS). NFDS arises when individuals of a rare morph experiences a higher fitness than those of a more common type [4]. Over generations, and in absence of other mechanisms, NFDS should lead to a balanced polymorphism, typically with limited fluctuations along an equilibrium frequency of the involved morphs [5-7]. Classic examples of NFDS include coexistence of different colour morphs, to gain access to mates [8], to challenge predators [9] and to lower sexual conflict intensity [10]. Although the idea of NFDS has been appreciated for decades [4,11], relatively few empirical studies have tested the validity of this concept under natural, unmanipulated field conditions. Especially rare are studies which relate natural geographical variation in morph frequencies, a putative selective agent and fitness parameters of the involved morphs with one another [7,10,12-14].

Sex-limited polymorphisms represent excellent model systems to study the nature of diversifying selection and consequently they have been subject to a variety of experimental [15-17] and theoretical [18,19] studies. Particularly popular are studies on male polymorphisms, whose maintenance tend to be explained by a fitness advantage to the rare morph relative to the common phenotypes in the competition over mates (e.g. sneakers do better when territorials predominate; reviewed by Oliveira et al. [8]). Over the last few decades, however, it has become clear that polymorphisms restricted to the female sex are more common in nature than previously thought [20,21]. Yet the underlying mechanisms that maintain phenotypic and genetic variation within females remain unresolved in many cases. Female polymorphisms are often considered to have evolved as a counter adaptation to reduce costs of harassment imposed by mate-searching males; e.g. butterflies [22], diving beetles [19], African bat bugs [23], damselflies [24]. The wider context of this proposed mechanism is sexual conflict over optimal mating rate. Evidently, females need males to fulfil their reproductive needs. However, obtrusive males may reduce female fitness by exceeding the females’ optimal number of matings [25-27]. Mate searching males are considered to face fewer cognitive challenges when confronted with only one, rather than multiple female phenotypes coexisting within populations [28], some of which may appear like males (i.e. andromorphs [29,30]). Therefore on a phenotypic level, females may experience diversifying sexual selection to avoid sexual harassment [31].

If the above arguments hold, then it is highly likely that female polymorphism is maintained by NFDS driven by social interactions between sexes. In much the same way that predators form a search image for the most common cryptic prey type [9], increased male sexual interest has been observed towards the most common female morph in a given population [19,32-34]. Recent studies showed an inverse relationship between morph-specific fecundity and morph-specific frequency in the population and suggested male harassment as most likely selective agent [7,10]. Although quantification of this selective agent has been understudied in past studies, support may come from density-dependent effects on fitness. Indeed, the overall intensity of male harassment may rise with male density, either absolute or relative to female density (i.e. operational sex ratio), because male–female interactions occur more frequently under these conditions [35-37]. Thus a thorough investigation of NFDS in female polymorphic systems entails evaluating the role of female morph frequency together with male densities on fitness related parameters [17], which forms the first aim of the current study.

In addition, sex-limited polymorphisms are frequently considered as alternative reproductive tactics (ARTs), i.e. a discontinuous set of selected traits to maximize reproductive output in two or more alternative ways [8]. Although repeatedly studied in males, recent observations in several species of owl [1], lizard [15,38] and insect [21,39,40] collectively indicate that female morphs may also represent ARTs. For example in the polymorphic lizard *Uta stansburiana*[15], combined density-dependent and negative frequency-dependent interactions among conspecifics determine the relative success of orange (produce many small eggs, *r*-strategy) and yellow (fewer but larger eggs, *K*-strategy) throated females. Female morphs may therefore differently allocate resources towards fitness related traits, potentially resulting in trade-offs among life-history and/or physiological traits. Although the majority of the studies cited above provide a new promising research avenue, they should be treated with caution, since many of them are performed with limited spatial replicates, without temporal replicates and/or with small sample sizes within populations. This makes it difficult to reach firm conclusions as to whether female morphs represent ARTs, especially in spatial and temporal heterogeneous environments.

In this study, we examine morph-specific variation in fecundity (i.e. egg number, egg mass, clutch mass and relative body mass) under natural conditions in multiple years and across six populations, which show extreme variation in morph frequencies. The aim of this study is to evaluate two hypotheses which try to explain maintenance of female polymorphism. Based on the first hypothesis we expect that relative male density, as a proxy for intensity of male harassment [35-37] and meanwhile the suggested selective force, negatively affects overall female fecundity. In doing so, we simultaneously test whether a frequency-dependent fecundity advantage exists for the rare female morph due to a lower positive frequency-dependent male detection rate [7,10,34]. Testing the second hypothesis, we explore whether female morphs exhibit ARTs in which resources are allocated differently into qualitative (egg mass) or quantitative (egg number) fecundity traits [15,38,39] and meanwhile account for potential spatial and temporal variation.

## Methods

### Model system

Female polymorphism has been observed in more than a hundred damselfly and dragonfly species [41]. Phenotypic ratios in laboratory cross experiments are consistent with the hypothesis that this polymorphism is genetically controlled by a single autosomal locus, with a number of alleles equal to the number of female morphs; reviewed by [42]. Large geographic variation in frequencies and densities of males and female morphs has been described in several species [43-45], which allows us to investigate the role of social interactions in maintaining sex-limited polymorphisms. We studied the common North American damselfly, *Nehalennia irene*, for which male harassment estimates towards female morphs has been shown to vary in a positive frequency-dependent manner [34, Bots J, Iserbyt A, Hammers M, Van Gossum H, Sherratt TN: Frequency-dependent sexual selection in two intra-specific mimicry systems, in preparation]. *Nehalennia irene* is not an endangered nor a protected species (see COSEWIC, federal government Canada) and therefore our research complies with the Convention on Biological Diversity and the Convention on the Trade in Endangered Species of Wild Fauna and Flora. It is a small non-territorial species, which inhabits marshy or boggy waters [46] and exhibits a discrete polymorphism restricted to the female sex. Female morphs are easily classified into andromorphs or gynomorphs based on their body coloration. Thus, while andromorph females resemble the conspecific male’s body blue coloration and melanin pattern [47,48], gynomorph females have distinctive yellowish lateral thorax sides and a less conspicuous abdominal melanin pattern; for colour figures see [49], for pictures see [50]. The species has one generation per year, with reproduction occurring between early June and mid-August. After locating a potential mate, a male will attempt grasping the individual in the so-called tandem formation, i.e. when the male succeeds in attaching his anal appendages to the individual’s prothorax [51]. This tandem formation can last several hours (AI, personal observation). If receptive, the female cooperates by bending her abdomen towards the male’s secondary genitalia (2nd abdominal segment) to form a ‘copulation wheel’ [51]. Very little additional information on reproductive biology is known for this species, but we expect that similar to related damselfly species, ovarial follicles of adult females can develop within one or a few days into roughly two hundred mature eggs [52]. In *N. irene*, females lay eggs in floating pieces of dead plant material while the female is tandem-guarded by her last successful male [53]. Several clutches of eggs are laid throughout a female’s lifetime [51].

### Study sites and sample procedures

Our previous work with *N. irene* indicated large spatial, yet limited temporal variation in female morph frequencies among populations. Specifically, andromorph frequencies range from 0 to >90% throughout the species’ distribution range over Canada [42,54]. For our current aims we selected six study populations that differed significantly in social conditions (see Table 1). Frequency and density estimates were obtained in a manner similar to that described in Van Gossum *et al.*[54]. In short, individuals were randomly captured with an insect net while walking slowly through the reproductive area, sweeping eight-shaped figures and recording the time elapsed. All caught males, andromorphs and gynomorphs were counted and marked with a permanent marker prior to release to avoid multiple counts. Andromorph frequency (proportion andromorph females), operational sex ratio (OSR, proportion males relative to females in the reproductive zone) and male density (number of males caught per minute) were calculated and collectively quantify the social environment. Calculating these parameters including or excluding immature individuals gave very similar results, see also [42]. Hence, we here use data based on mature, thus reproductively active, individuals given that the aim of the current study deals with sexually active individuals. OSR and male density can be used as a proxy for overall male harassment in a given population [34,35,55,56]. Each of the six populations were monitored during the reproductive season over three consecutive years (2007–2009).

**Table 1:** Geographic location of the studied populations and key estimates of the social environment

**Population**	**Lat / Long**	**Afreq**	**OSR**	**Mdens**
Barb’s Marsh (ON)	44°31′/ -76°22′	0.06 ± 0.03	2.33 ± 0.41	18.2 ± 8.7
Jack’s Marsh (ON)	44°34′/ -76°20′	0.07 ± 0.05	3.01 ± 0.28	29.2 ± 16.7
Otter Marsh (ON)	44°33′/ -76°22′	0.34 ± 0.11	3.38 ± 0.29	22.2 ± 11.0
Quebec City (QU)	46°46′/ -70°58′	0.70 ± 0.10	1.55 ± 0.52	6.4 ± 1.1
Summit Lake (BC)	54°10′/ -122°41′	0.94 ± 0.02	1.34 ± 0.20	9.5 ± 3.7
Airpark Road (BC)	54°00′/ -123°02′	0.94 ± 0.01	1.56 ± 0.18	8.8 ± 2.8

Estimates of andromorph frequency (Afreq), operational sex ratio (OSR) and male density (Mdens) per population and province (Ontario, Quebec and British Columbia), averaged across three study years (mean ± 1SE). An extended table with detailed information on sample sizes per population and per year is given in (Additional file 1).

Sample collection was carried out on several days throughout the reproductive season (mean ± SE: 4 ± 0.5 sample days; see Additional file 1) always between 9 am and 3 pm. This is the period before the majority of females start to oviposit (AI, personal observations). Andromorphs and gynomorphs were collected in an alternating manner to maintain a balanced design and to control for potential diurnal variation in egg number. We aimed to collect 25 adult andromorphs and 25 gynomorphs in each population for each investigated year. Measurements for relative body mass (see below) were performed in all three years (N = 43 ± 3 females per population and year; total N = 772) and the three other fecundity estimates were investigated in two successive years (N = 44 ± 3 females per population and year; total N = 547). For more detailed sample sizes per population and per year, (see Additional file 1). Mating status at the moment of capture was noted, i.e. being single or mating (i.e. involved in tandem or copulation). All individuals were stored for further measurements in 95% ethanol immediately after capture.

### Fecundity estimates

First, an individual was placed on a sheet of absorbent paper for exactly two minutes to allow standardised evaporation and absorption of most of the ethanol. Then it was weighted on a Kern & Sohn GmbH 870 balance (accuracy 0.1 mg). Next, a digital picture was taken (Nikon D70/Tamron macro lens 90 mm 1:2.8) of the right hind wing. Using ImageJ 1.38x [57], wing length was measured from the second antenodal cross vein to the stigma; see [50] for more details. Residuals of body mass were calculated by regressing body mass against wing length and were used as a measure for relative body mass (RBM). Positive values indicate relative heavy individuals for a given wing size, while negative values indicate relatively light individuals. RBM is not only considered an estimate for body condition, it is also suggested to increase with female fecundity in various insect taxa [58]. Hence, RBM is here treated as a coarse measure of overall fecundity.

Dissection of specimens was performed under a Leica MZ 12.5 stereomicroscope. Abdominal sternites were removed and fifty developed eggs were isolated on a pre-weighted aluminum foil. This high number of eggs was chosen to account for potential variation in weight among eggs of the same clutch. Eggs were then dried in an incubator (Binder – APT.line™) at 60°C for 12 h and weighted on a Sartorius SE2F balance (accuracy 1 μg). Average dry weight of a single egg was calculated and further considered as a measure of egg quality since more nutrients are expected in heavier eggs [59,60]. The total number of developed eggs was also counted for each specimen, and considered a quantitative measure of fecundity. Multiplying egg number with egg mass gave clutch mass as a comprehensive measure of fecundity (i.e. quality*quantity).

By measuring twice a set of randomly chosen individuals among populations and years, the repeatability of our measures could be evaluated. Repeatability was calculated as the proportion of the variation between individuals to the total variation, i.e. between and within individuals [61]. A limited measurement error was observed: body mass (R = 0.90, N = 106), wing length (R = 0.96, N = 136), egg mass (R = 0.78, N = 46), and egg number (R = 1.00, N = 66).

### Statistical analyses

To evaluate our first hypothesis, we initially tested for statistical dependence in our estimates of the social environment. Using single regressions and adding study population as a repeated measure, andromorph frequency, OSR and male density appeared not significantly related to one another (all P ≥ 0.17 and Spearman R^2^ ≤ 0.32). This allowed us to simultaneously test the predictive value of these parameters, along with female morph and their morph-specific interactions (see Table 1) in the same mixed ANCOVA models. Four such models were fitted to investigate variation in the fecundity measures, while controlling for spatial and temporal variation, as well as potential differences between mated and single females by adding study population, year and mating status as random variables.

With regards to our second hypothesis, we tested whether both morphs differentially invested in qualitative (egg mass) or quantitative (egg number) reproductive traits. In doing so, we fitted an ANCOVA model with egg number treated as response variable and female morph, egg mass and their interactions as explanatory variables. A significant effect of the interaction would suggest a morph-specific trade-off among both reproductive traits. Meanwhile, we controlled for annual and spatial variation, as well as potential effects of mating status in this analysis by adding year, study population and mating status as random variables to the models. All analyses were performed in SAS 9.2 (SAS Institute Inc, Cary, NC, USA).

## Results

**Figure 1:**
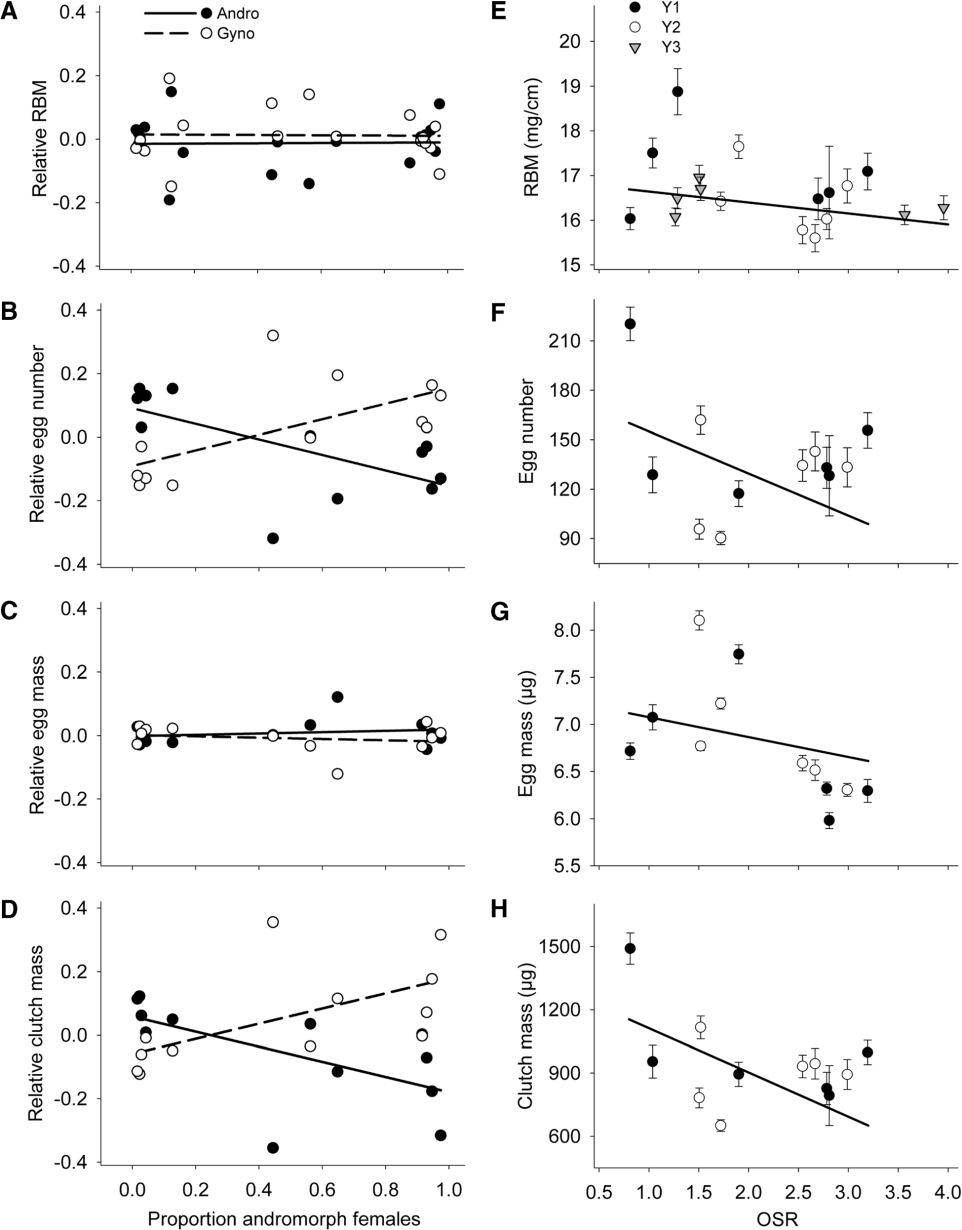
**Variation in fecundity traits of the damselfly *N. irene*.** Panel **A-D**: Graphical interpretation of the female morph by andromorph frequency interaction. Relative fecundity for each measurement is calculated in each population and year as RF_A_ = ln(F_A_/F_G_) for andromorphs and RF_G_ = ln(F_G_/F_A_) for gynomorphs, similar to [7]. Values above and below 0 indicate respectively, higher fecundity for andromorph (black symbols, solid line), relative to gynomorphs (white symbols, dashed line) females. Panel **E-H**: Decrease in fecundity estimates with operational sex ratio (OSR). Mean (± 1SE) values are given for each population and each year. Black circles, white circles and gray triangles represent fecundity in the respective successive years (Y1, Y2 and Y3). Regression curves are based on the parameter estimates of the ANCOVA models, thus including geographical and temporal dependency of the data points.

With regards to our first hypothesis, both egg number (F_1,534_ = 4.2; P = 0.04) and clutch mass (F_1,515_ = 5.6; P = 0.02) varied with population morph frequency and in opposite directions for both female morphs (see Morph*Afreq, Table 2), which provides support for NFDS on female fecundity. Specifically, andromorph females store 8.7% more eggs and have 5.4% higher clutch mass when rare, compared with gynomorphs (Figure 1B,D). This fecundity advantage for andromorphs gradually switched with rising andromorph frequencies towards a reverse situation with higher fecundity for gynomorphs when rare (14.4% and 16.5% difference in egg number and egg mass, respectively). A morph-specific and frequency-dependent effect was not found for egg mass and relative body mass (Table 2; Figure 1A,C). However, all four investigated fecundity estimates significantly decreased with operational sex ratio (P ≤ 0.03; Figure 1E-H), which had similar effects on andromorphs and gynomorphs (see Morph*OSR, Table 2). Male density on the other hand, had no effect on both overall fecundity measures, neither as a main effect (P ≥ 0.40), nor as an interaction with female morph (P ≥ 0.16), see Table 2. As body size may vary with latitude reviewed by [62] and therefore potentially may influence egg number, we performed separate analyses which also included wing length. However, none of these additional analyses altered the outcome of the analyses presented in Table 2.

**Table 2:** Results of the ANCOVA analyses explaining variation in four key fecundity estimates

	**Effect**	**DF**	**F**	**P**
-Relative body mass				
	Morph	761	0.78	0.38
	Afreq	758	0.96	0.33
	OSR	762	4.7	**0.031**
	Mdens	759	0.72	0.40
	Morph*Afreq	756	0	0.98
	Morph*OSR	760	1.1	0.29
	Morph*Mdens	757	1.99	0.16
-Egg mass				
	Morph	516	1.2	0.27
	Afreq	517	9.57	**0.002**
	OSR	517	20.49	**<.0001**
	Mdens	513	0.01	0.93
	Morph*Afreq	514	0.11	0.75
	Morph*OSR	515	0.82	0.37
	Morph*Mdens	512	0	0.98
-Egg number				
	Morph	534	1.95	0.16
	Afreq	534	8.48	**0.004**
	OSR	534	19.22	**<.0001**
	Mdens	532	0.08	0.78
	Morph*Afreq	534	4.22	**0.040**
	Morph*OSR	533	0.12	0.73
	Morph*Mdens	531	0.18	0.67
-Clutch mass				
	Morph	515	2.2	0.13
	Afreq	515	4.97	**0.026**
	OSR	515	33.85	**<.0001**
	Mdens	514	0.52	0.47
	Morph*Afreq	515	5.59	**0.019**
	Morph*OSR	513	0.44	0.5083
	Morph*Mdens	512	0.0	0.9976

Explanatory variables include female morph frequencies (Afreq), male density (Mdens) and operational sex ratio (OSR). All four analyses are controlled for variation among populations, years and mating status (see methods). The asterisks (*) indicate tested interactions between two main effects. Numerator degrees of freedom is 1 in all cases, DF in the table refers to the denominator degrees of freedom. Note that egg mass, egg number and relative clutch mass were measured in two successive years and body mass in three subsequent years. Significant results are indicated in bold.

**Figure 2:**
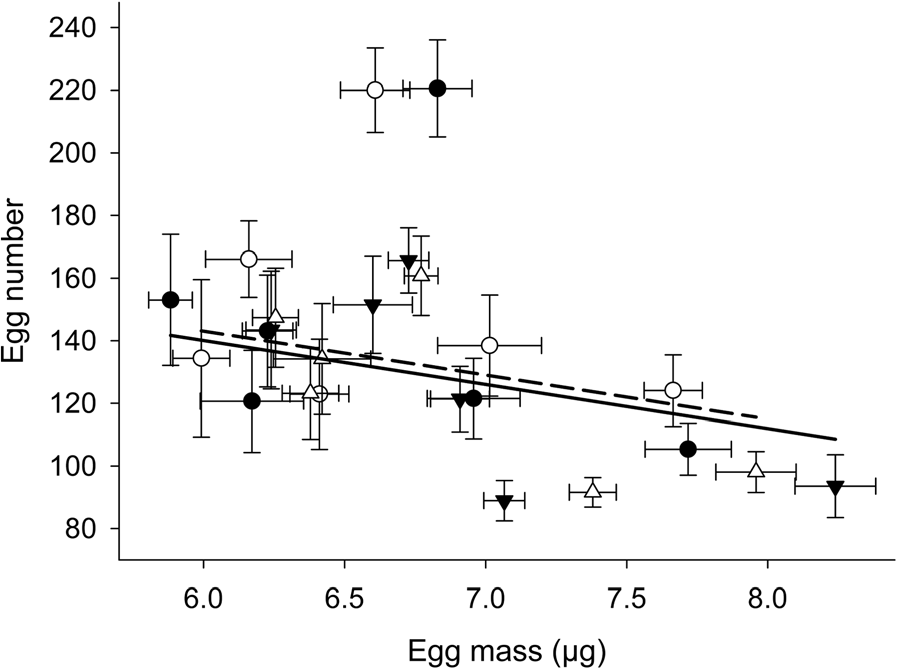
**Graphical interpretation of the quantity-quality trade-off.** Decrease in egg number with egg mass for andromorphs (black symbols, solid line) and gynomorphs (white symbols, dashed line). Mean (± 1SE) values of both fecundity traits are given for each study population and for both investigated years. Circles and triangles represent, respectively, fecundity in the first and the subsequent year. Regression curves are based on the parameter estimates of the ANCOVA model, thus including geographical and temporal dependency of the data points.

Finally, a negative correlation was observed between egg mass and egg number (F_1,518_ = 10.2, P = 0.002). This reproductive trade-off was observed within all populations, except in Quebec City (see Additional file 2). Interestingly, our results also show a trade-off across populations, in which females from different populations seem to invest more into either egg number or egg mass (Figure 2). However, the resource allocation towards these traits did not differ between female morphs (egg mass*morph: F_1,516_ = 0.43, P = 0.51; Figure 2). Finally, when controlling for spatial and temporal variation, female morphs did not differ in egg number (F_1,537_ = 0.08, P = 0.78) or egg mass (F_1,518_ = 0.99, P = 0.32).

## Discussion

Our study provides some of the first empirical evidence for NFDS on female fecundity in natural conditions and as such provides an important key to understanding the maintenance of intra-sexual phenotypic and genetic variation in this species. Our conclusion was influenced by our observation of a significant inverse relationship between all four of our fecundity measures and relative male density (OSR) as an index of male harassment rate. Indeed, obtrusive males may reduce female fitness in a variety of ways [25,26,63] ranging from physical damage to inhibiting foraging success, resulting in suboptimal fecundity; e.g. bees [64], damselflies [52]. Thus our reported relationships are consistent with the idea that male sexual harassment comes with a fitness costs in females and therefore acts as a major selective force in this study system; see also [55]. Second, we show that the rare female morph has a higher egg load relative to the common one. Clutch mass, as our comprehensive fecundity measure, shows a similar frequency-dependent relationship and is most likely driven by the pattern in egg load. Intriguingly recent work with *N. irene*, involving exactly the same populations and the same years as the current study, clearly indicated that males prefer to mate with the most common female morph [34, Bots J, Iserbyt A, Hammers M, Van Gossum H, Sherratt TN: Frequency-dependent sexual selection in two intra-specific mimicry systems, in preparation]. All of the above observations collectively support our first hypothesis that positive frequency-dependent male harassment translates into the currently presented negative frequency-dependent patterns in female morph fecundity. Together with the work on polymorphic lizards [12], diving beetles [19] and other damselfly species [7,10] our work emphasises the importance of costly frequency -dependent social interactions as a balancing mechanism to explain intra-specific polymorphisms.

Theoretical and empirical studies indicate that in absence of other mechanisms, NFDS could over generations lead to limited temporal frequency fluctuations along an equilibrium frequency; e.g. fishes [5], damselflies [7]. Morph frequencies vary little over the years in *N. irene* (0 - 25%) compared to the very large spatial variation (0 - ~100% [43], AI unpublished results). In fact, such large spatial variation has now been reported repeatedly in polymorphic damselflies and often resembles a geographical cline (*Ischnura elegans*[44]*, I. senegalensis*[45]*, N. irene*[54]*, Megalagrion calliphya*[65]). A single equilibrium frequency is thus clearly not reached. This may indicate that besides NFDS operating within populations, additional mechanisms may influence the currently observed population morph frequencies. An obvious suggestion is that divergent selection or gene-by-environment interactions [45] affect the precise equilibrium, favouring certain morphs under a given set of ecological conditions such as particular densities of con- and heterospecific damselflies, differences in climate, predation rate or parasite load among populations. In addition, historical and present-day stochastic mechanisms have been proposed as well to explain the observed large geographic frequency variation, at least in some parts of a species’ distribution range [66,67]. Thus, although NFDS on female fecundity appears to be a key balancing mechanism operating within populations, the importance of additional mechanisms are still under debate and should deserve more attention in future research to explain this natural phenomenon thoroughly.

Interestingly, the fecundity advantage for andromorphs is much smaller when rare compared to gynomorphs, indicating an asymmetry in this relationship. This is surprising since it can be expected that andromorphs experience lower harassment rates and associated costs due to their male-like appearance, especially in populations where they are the rare morph [29,30,68,69]. Previous work further indicated that female phenotypic appearance varies with the population morph frequency in this system [51]. Indeed, andromorphs differ overall in body size and shape from gynomorphs [70,71]. However when common, andromorphs resemble the smaller conspecific male more closely than gynomorphs, which is consistent with theory explaining imperfect mimicry [30,72]. In addition to direct effects of male harassment (discussed above), it is likely that female fecundity is also shaped by morphological constraints, with smaller or slender females being limited in the number of eggs they can store; see also *Ischnura elegans*[33]. Thus, although male-like appearance of andromorphs may be beneficial in terms of lower detection rate by harassing males, mimicry may on the other hand come along with a fecundity cost due to allometric associations. The interplay between costs and benefits of mimicry may perhaps explain the asymmetry in our fecundity relationship. Taken all together, present-day patterns in female morph fecundity may relate to direct effects of costly male harassment, perhaps combined with indirect consequences of long-term selection that altered female morph morphology.

Our work also highlights a resource allocation trade-off, in which females invest in either quality (egg mass) or quantity (egg number). This trade-off not only holds within populations, but also differs among populations and may perhaps relate to the inhospitable aquatic environment of the larvae in terms of predation [73]. Odonate larvae are generalist predators that interact aggressively towards conspecifics or heterospecifics, leading to high rates of cannibalism [74-76]. Hatching from heavier eggs, resulting in larger larvae may thus be more advantageous in this (geographically heterogeneous [77]) competitive environment. Furthermore, we observed increasing egg mass and decreasing egg number towards Northern latitudes. It has been suggested before that body size may increase with latitude due to temperature related physical constraints of growth and development (Bergmann’s rule, reviewed by [62]), a general pattern which is also confirmed in damselflies [45,50]. Taken together, investment in heavier eggs may depend on several ecological variables, including abiotic conditions [78] and degree of the competitive environment [73], but will most likely come at the expense of producing numerous eggs.

This reproductive trade-off, however, did not differ between morphs, which contrasts with hypothesis two and earlier observations in polymorphic birds [1] and lizards [15,38]. These former studies indicated that female morphs may differently allocate resources among life-history traits. The current result also contrasts with recently reported alternative physiological optima in *N. irene*[40]. Specifically, this latter study showed that andromorphs, compared to gynomorphs, invest more in traits related to immune function and less in flight muscles. In fact, numerous studies with female polymorphic damselflies investigated different life-history components, physiological traits (summarized by [79]) and morph-specific behavioural strategies to cope with male harassment [33,80,81]. Mixed results have been reported previously, but often in favour of the hypothesis that female damselfly morphs represent ARTs in order to escape from excessive male harassment. The combination of traits that differ between the female morphs may be context dependent and/or species specific. Therefore, we suggest that future studies on ARTs focus on an integrated set of behavioural and fitness related traits in different social contexts, and perhaps quantify life-time reproductive success by means of molecular tools [82].

## Conclusion

In conclusion, we provide some of the first empirical evidence collected in natural populations with extreme variation in morph frequencies demonstrating NFDS on female fecundity. As fecundity is a key component of fitness, our results explain an important part of the mechanism maintaining intra-sexual polymorphisms. Frequency-dependent male sexual harassment may well be the driving force of this pattern in our study system, either directly or indirectly affecting egg number and clutch mass of female morphs in a frequency-dependent way. We also show that female morphs do not differently allocate resources into quantitative or qualitative fecundity traits, although this does not exclude the potential for alternative reproductive tactics in this system. As Oliveira et al. [8] argues, studies on female ARTs are largely understudied and should deserve more attention in future research.

## Abbreviations

Afreq: Andromorph frequency; ARTs: Alternative reproductive tactics; Mdens: Male density; NFDS: Negative frequency-dependent selection; OSR: Operational sex ratio; RBM: Relative body mass.

## Competing interests

The authors declare that they have no competing interests.

## Authors’ contributions

All authors participated in designing the study, performing field work, providing financial support and drafting this manuscript. In addition, AI performed all dissections, statistically analysed the dataset and created all graphs. All authors read and approved the final manuscript.

## Supplementary Material

Additional file 1 **Detailed overview of sample dates (N **_**sw**_**), key estimates of the social environment and female morph fecundity.** Click here for file

Additional file 2 Quantity-quality trade-off within each study population. Click here for file
