# Supplementary material for: Negative frequency-dependent selection or alternative reproductive tactics: maintenance of female polymorphism in natural populations
Source: BMC Evol Biol. 2013 Jul 3;13:139. doi: 10.1186/1471-2148-13-139 (PMC3704290; doi:10.1186/1471-2148-13-139)
Supplement: Additional file 1 — Detailed overview of sample dates (N sw), key estimates of the social environment and female morph fecundity. [file 1471-2148-13-139-S1.docx]

**Additional file 1 – Detailed overview of sample dates (N _sw_), key estimates of the social environment and female morph fecundity.**

Estimated andromorph frequency (Afreq), operational sex ratio (OSR) and male density (Mdens) in given for each population and year. These estimates are based on the total number of individuals counted during sweep sessions (N_sw_). Mean (± 1SE) relative body mass (RBM, mg/cm), egg number, egg mass (µg) and clutch mass (µg) is given for both for both female morphs in each investigated population and year. N_A_ (andromorph) and N_G_ (gynomorphs) refer to the sample size used theretofore. Note that RBM is measured in three study years and the other three fecundity estimates are quantified in two successive years.

| **Population** | **Year** | **Date** | **N_sd_** | **N_sw_** | **Afreq** | **OSR** | **Mdens** | **Andromorph** | | | | |  | **Gynomorph** | | | | |
| --- | --- | --- | --- | --- | --- | --- | --- | --- | --- | --- | --- | --- | --- | --- | --- | --- | --- | --- |
|  |  |  |  |  |  |  |  | **N_A_** | **RBM** | **Egg number** | **Egg mass** | **Clutch mass** |  | **N_G_** | **RBM** | **Egg number** | **Egg mass** | **Clutch mass** |
| Barb's Marsh | 2007 | 11-15/6 | 5 | 122 | 0.12 | 2.7 | 9.2 | 15 | 7.72±0.24 | - | - | - |  | 18 | 9.34±0.23 | - | - | - |
| Barb's Marsh | 2008 | 24/6 - 4/7 | 7 | 333 | 0.02 | 2.8 | 9.9 | 25 | 8.31±0.16 | 143.1±17.9 | 6.23±0.09 | 877.4±107.3 |  | 25 | 8.14±0.20 | 122.9±17.6 | 6.41±0.10 | 776.3±110.6 |
| Barb's Marsh | 2009 | 16-19/6 | 3 | 529 | 0.03 | 1.5 | 35.7 | 25 | 8.96±0.13 | 163.4±11.3 | 6.77±0.06 | 1155.3±58.7 |  | 28 | 8.92±0.17 | 160.8±12.6 | 6.77±0.07 | 1087.2±85.3 |
| Jack's Marsh | 2007 | 3/6-2/7 | 6 | 179 | 0.04 | 2.8 | 9.4 | 15 | 7.73±0.14 | 146.8±32.6 | 6.01±0.09 | 875.0±188.1 |  | 15 | 7.45±0.15 | 109.5±37.2 | 5.95±0.15 | 700.0±226.9 |
| Jack's Marsh | 2008 | 26/6-7/7 | 9 | 231 | 0.02 | 2.7 | 15.8 | 25 | 8.21±0.28 | 151.5±15.6 | 6.60±0.14 | 995.9±105.5 |  | 25 | 7.97±0.18 | 134.2±17.7 | 6.42±0.17 | 888.8±99.4 |
| Jack's Marsh | 2009 | 21-22/6 | 2 | 388 | 0.16 | 3.6 | 62.4 | 25 | 8.32±0.16 | - | - | - |  | 25 | 8.69±0.17 | - | - | - |
| Otter Marsh | 2007 | 12-16/7 | 3 | 151 | 0.44 | 3.2 | 9.4 | 26 | 8.29±0.35 | 139.7±19.6 | 6.52±0.19 | 910.6±115.1 |  | 27 | 9.28±0.25 | 166.1±12.2 | 6.16±0.15 | 1050.1±62.2 |
| Otter Marsh | 2008 | 8-11/7 | 3 | 1129 | 0.13 | 3.0 | 13.1 | 25 | 9.14±0.26 | 143.4±18.8 | 6.24±0.09 | 913.9±107.5 |  | 25 | 7.87±0.17 | 123.2±14.6 | 6.38±0.10 | 869.6±92.4 |
| Otter Marsh | 2009 | 29-30/6 | 2 | 332 | 0.46 | 4.0 | 44.1 | 25 | 8.34±0.19 | - | - | - |  | 25 | 8.41±0.21 | - | - | - |
| Quebec City | 2007 | 10/7 | 1 | 116 | 0.56 | 0.8 | 8.2 | 27 | 7.89±0.13 | 220.6±15.4 | 6.83±0.12 | 1517.0±113.1 |  | 28 | 9.08±0.27 | 200.0±13.5 | 6.61±0.12 | 1464.4±98.7 |
| Quebec City | 2008 | 15-19/7 | 3 | 131 | 0.65 | 2.5 | 4.5 | 25 | 8.05±0.20 | 121.4±10.5 | 6.91±0.10 | 864.1±60.6 |  | 25 | 8.12±0.18 | 147.4±15.9 | 6.26±0.08 | 1002.1±88.9 |
| Quebec City | 2009 | 23-24/6 | 2 | 96 | 0.88 | 1.3 | 6.5 | 25 | 8.23±0.21 | - | - | - |  | 25 | 8.87±0.18 | - | - | - |
| Summit Lake | 2007 | 26/6-4/7 | 7 | 161 | 0.97 | 1.0 | 4.1 | 27 | 9.42±0.21 | 121.5±12.8 | 6.96±0.16 | 840.1±89.1 |  | 25 | 8.43±0.21 | 141.6±20.1 | 7.32±0.22 | 1190.1±134.6 |
| Summit Lake | 2008 | 14-15/7 | 2 | 117 | 0.93 | 1.7 | 7.9 | 25 | 9.25±0.18 | 89.0±6.5 | 7.07±0.07 | 627.1±44.6 |  | 25 | 9.13±0.14 | 91.6±4.7 | 7.38±0.08 | 673.6±32.9 |
| Summit Lake | 2009 | 5-6/7 | 2 | 265 | 0.92 | 1.3 | 16.5 | 25 | 8.88±0.17 | - | - | - |  | 29 | 8.92±0.15 | - | - | - |
| Airpark Road | 2007 | 20/6 - 4/7 | 3 | 119 | 0.96 | 1.3 | 3.2 | 25 | 9.92±0.21 | - | - | - |  | 15 | 10.32±0.37 | - | - | - |
| Airpark Road | 2008 | 29/6 - 13/7 | 5 | 438 | 0.95 | 1.9 | 10.6 | 25 | 9.84±0.18 | 108.4±8.9 | 7.70±0.15 | 812.7±65.5 |  | 25 | 9.57±0.17 | 128.9±13.6 | 7.80±0.13 | 995.3±97.1 |
| Airpark Road | 2009 | 11-16/7 | 6 | 268 | 0.92 | 1.5 | 12.5 | 28 | 9.60±0.16 | 93.6±10.1 | 8.24±0.14 | 783.6±77.1 |  | 25 | 9.54±0.15 | 98.1±6.5 | 7.96±0.14 | 781.5±53.4 |
