# Supplementary material for: Negative frequency-dependent selection or alternative reproductive tactics: maintenance of female polymorphism in natural populations
Source: BMC Evol Biol. 2013 Jul 3;13:139. doi: 10.1186/1471-2148-13-139 (PMC3704290; doi:10.1186/1471-2148-13-139)
Supplement: Additional file 2 — Quantity-quality trade-off within each study population. [file 1471-2148-13-139-S2.docx]

**Additional file 2 – Quantity-quality trade-off within each study population.**

Variation in egg mass and egg number is given separately for andromorphs (black symbols, solid line) and gynomorphs (white symbols, dashed line).
